# Infrared Plasmonic Biosensor with Tetrahedral DNA Nanostructure as Carriers for Label‐Free and Ultrasensitive Detection of *miR‐155*

**DOI:** 10.1002/advs.202100583

**Published:** 2021-06-21

**Authors:** Xindan Hui, Cheng Yang, Dongxiao Li, Xianming He, He Huang, Hong Zhou, Ming Chen, Chengkuo Lee, Xiaojing Mu

**Affiliations:** ^1^ Key Laboratory of Optoelectronic Technology and Systems Ministry of Education International R&D Center of Micro‐Nano Systems and New Materials Technology Chongqing University Chongqing 400044 P. R. China; ^2^ Department of Clinical Laboratory Southwest Hospital Third Military Medical University (Army Medical University) Chongqing 400038 China; ^3^ Department of Electrical and Computer Engineering Center for Intelligent Sensors and MEMS (CISM) NUS Graduate School for Integrative Sciences and Engineering National University of Singapore Singapore 117576 Singapore

**Keywords:** biosensors, infrared spectroscopy, metamaterial absorbers, surface‐enhanced infrared absorption

## Abstract

MicroRNAs play an important role in early development, cell proliferation, apoptosis, and cell death, and are aberrantly expressed in many types of cancers. To understand their function and diagnose cancer at an early stage, it is crucial to quantitatively detect microRNA without invasive labels. Here, a plasmonic biosensor based on surface‐enhanced infrared absorption (SEIRA) for rapid, label‐free, and ultrasensitive detection of *miR‐155* is reported. This technology leverages metamaterial perfect absorbers stimulating the SEIRA effect to provide up to 1000‐fold near‐field intensity enhancement over the microRNA fingerprint spectral bands. Additionally, it is discovered that the limit of detection (LOD) of the biosensor can be greatly improved by using tetrahedral DNA nanostructure (TDN) as carriers. By using near‐field enhancement of SEIRA and specific binding of TDN, the biosensor achieves label‐free detection of *miR‐155* with a high sensitivity of 1.162% pm ^−1^ and an excellent LOD of 100 × 10^−15^ m. The LOD is about 5000 times lower than that using DNA single strand as probes and about 100 times lower than that of the fluorescence detection method. This work can not only provide a powerful diagnosis tool for the microRNAs detection but also gain new insights into the field of label‐free and ultrasensitive SEIRA‐based biosensing.

## Graphical abstract

The detection of diagnostic and prognostic biomarkers for cancer is of great significance in clinical medicine. A plasmonic biosensor based on surface‐enhanced infrared absorption is demonstrated for rapid, label‐free, and ultrasensitive detection of cancer biomarker *miR‐155*. It is discovered that the limit of detection of the biosensor can be greatly broken by using tetrahedral DNA nanostructure as carriers.

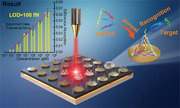

## 1 Introduction

The mortality of cancer remains an important cause of death worldwide, as most clinical signs of cancer appear in the late stages, when treatment can only alleviate suffering but not cure the disease.^[^ ^1^ ^]^ Thus, diagnosis of early cancer contributes to more efficient treatment and prolongs the survival of patients.^[^ ^2^ ^]^ MicroRNAs are a type of endogenous, noncoding, single‐stranded small RNA molecule with a length of about 20–24 nucleotides that regulate the critical functions in various biological processes, such as early development, cell proliferation, apoptosis, and cell death.^[^ ^3^ ^]^ It is demonstrated by many studies that the microRNAs are abnormally expressed in a variety of cancers,^[^ ^4^ ^]^ including lung cancer,^[^ ^5^ ^]^ prostate cancer,^[^ ^6^ ^]^ pancreatic cancer,^[^ ^7^ ^]^ breast cancer,^[^ ^8^ ^]^ and colorectal cancer.^[^ ^9^ ^]^ Therefore, microRNAs are ideal potential diagnostic and prognostic biomarkers for the corresponding cancer detection.^[^ ^10^ ^]^ There are many methods for the detection of microRNAs, including fluorescent and electrochemical methods. For instance, Li et al. reported a DNAzyme‐based amplification method for detection of *miR‐155* with a concentration from 0.1 to 10 × 10^−9^ m and a detection limit of 44 × 10^−12^ m.^[^ ^11^ ^]^ Wang et al. proposed an electrochemistry biosensor based on the copper nanoclusters and triple amplification methods for ultrasensitive detection of *miR‐21* with a concentration from 10 × 10^−12^ to 0.1 × 10^−15^ m and a detection limit of 10 × 10^−18^ m.^[^ ^12^ ^]^ Although these studies provide effective ways to detect microRNAs, these methods require toxic markers or aptamers, resulting in tedious operations and time‐consuming detection procedures. Therefore, a new, simple, and inexpensive technology for rapid, label‐free, and ultrasensitive detection of microRNA still presents a challenge.

Many efforts have been invested for the development of label‐free and ultrasensitive detection methods, including mass‐loading methods, surface plasmon resonance methods, and spectroscopy. These methods have high sensitivity, but their selectivity depends entirely on the specific binding of the probe to the target molecule, which leads to defects in sensitivity to the external environment, including temperature, humidity, and other molecules interference. Infrared (IR) spectroscopy is a widely used spectral analysis technique.^[^ ^13^ ^]^ Because any molecular species has a set of vibration modes and the absorption caused by its vibration lies in the mid‐infrared range and forms a unique fingerprint,^[^ ^14^ ^]^ IR spectroscopy can detect these fingerprints and identify different biomolecules in a nondestructive and noninvasive way.^[^ ^15^ ^]^ As this method is based on the internal infrared vibration of the target molecule instead of relying on labels, it eliminates the interference from the label (such as fluorescence quenching). Despite these advantages, traditional IR methods suffer from poor sensitivity and high analytes consumption^[^ ^16^ ^]^ as it requires long optical interaction length to achieve µm level detection limit.^[^ ^17^ ^]^ Surface‐enhanced infrared absorption (SEIRA) spectroscopy is first reported by Hartstein et al. in 1980,^[^ ^18^ ^]^ and it is increasingly attracting interest because of its ability to overcome the sensitivity limit by using the near‐field coupling between plasmonic resonances and vibrational modes of target molecules.^[^ ^19^ ^]^ Apart from overcoming the sensitivity limit, SEIRA spectroscopy has shown great potential in identifying/characterizing various chemical^[^ ^20^ ^]^ and biological molecules,^[^ ^21^ ^]^ exploring reaction mechanisms, and monitoring kinetic processes.^[^ ^22^ ^]^ This inspired us to explore the possibility of microRNA detection using SEIRA technology and solve potential technical problems to make a significant breakthrough in rapid, label‐free, and ultrasensitive detection of microRNA. Although the use of SEIRA for sensing has been demonstrated by peers,^[^ ^23^ ^]^ ultrasensitive detection of miRNA by selecting appropriate probes and fabricating SERIA device with high performance is still a new and challenging task.

Here, we report a SEIRA‐based plasmonic biosensor with tetrahedral DNA nanostructure as carriers for rapid, label‐free, and ultrasensitive detection of *miR‐155*. The biosensor uses metamaterial perfect absorber (MPA) forming Fabry‐Perot optical cavity to provide up to 1000‐fold near‐field intensity enhancement over the *miR‐155* fingerprint spectral bands. Importantly, we discover the *miR‐155* limit of detection (LOD) of the SEIRA‐based biosensor can be greatly improved by using TDN as scaffolds, ≈5000 times lower than that using single‐stranded DNA (ssDNA) as probes. By exploiting the enhanced near‐field and sensitive TDN carriers, the SEIRA biosensor exhibits higher sensitivity than fluorescent method, but also has the advantages of simplicity, rapidity, stability, and reusability. We believe these findings provide a valuable toolkit for microRNA detection as well as gain new insights into the field of label‐free biosensing.

## 2 Results and Discussions

### 2.1 Detection Mechanism

**Figure 1:**
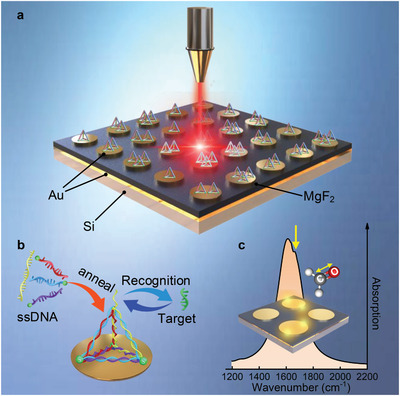
Illustration of the biosensor platform for detecting microRNA. a) Schematic view of the proposed biosensor platform. b) Schematic diagram of the construction of a tetrahedral DNA nanostructure (TDN). The TDN was assembled from three thiolated 54‐mer DNA fragments and one 82‐mer DNA fragment carrying the probe sequence. The TDN is deposited on the Au surface through physical adsorption, leaving a freestanding probe at the top to identify target. c) The absorber resonance position is designed to overlap with the vibration bond of C═O, achieving ultrasensitive sensing of *miR‐155*.

**Figure** **1** illustrates the mechanism of microRNA detection using the proposed plasmonic biosensor. The biosensor consists of a SEIRA‐based device and TDN carriers. The SEIRA‐based device provides enhanced near‐field, and the TDN functions as a probe to selectively capture microRNA (*miR‐155*) molecule by specific binding (Figure 1a). The selection of the carriers must be cautious because they determine the performance of the sensor, including selectivity, stability, and sensitivity. There are two choices of carrier: ssDNA and TDN. It is demonstrated by Fan group that the combination of TDN probes and electrochemical biosensors shows excellent detection performance.^[^ ^24^ ^]^ In addition, the distance between TDN probes is controllable to ensure probe orientation. The tetrahedral structure also has a certain thickness (≈6 nm), which provides a solution‐like reaction environment, thereby increasing the affinity.^[^ ^25^ ^]^ Therefore, TDN was chosen as the final probes combined with the SEIRA biosensor. TDN is composed of four ssDNA, of which the ends of the three short ssDNA are modified with thiol molecules to form the bottom in order to firmly anchor at the surface of the SEIRA platform, and another long ssDNA leaves a freestanding probe at the top to capture the target *miR‐155*, as shown in Figure 1b. The mid‐infrared spectrum of RNA is demonstrated in Note S1 (Supporting Information), existing vibrations and rotations of various molecular. Among them, the C═O bond around 1665 cm^−1^ has the strongest stretching vibration, which is selected as the target peak for the *miR‐155* detection.

There are many ways to stimulate SEIRA effect, such as nanowire,^[^ ^26^ ^]^ nanohole,^[^ ^27^ ^]^ nanosize gap,^[^ ^28^ ^]^ metasurfaces,^[^ ^29^ ^]^ nanocavities,^[^ ^30^ ^]^ and MPA.^[^ ^31^ ^]^ Among them, MPA has been considered as the good choice for SEIRA spectroscopy applications owing to its ability to perform the strong absorbance.^[^ ^32^ ^]^ The first MPA operating in the infrared regime was reported by Padilla group.^[^ ^33^ ^]^ Owing to its strong absorption and enhanced near‐field, MPA‐based SEIRA is widely used for biosensing.^[^ ^34^ ^]^ Besides, it discovered that the sensitivity of the MPA is increased by an order of magnitude when compared with the metamaterial surface working in transmission mode.^[^ ^33^ ^]^ Therefore, the MPA is chosen as the method to inspire SEIRA effect. The MPA consists of metallic nanoantennas on top, a dielectric spacer layer, and a thick Au sheet to form an oscillator. The free electrons on the metal surface will oscillate collectively under the excitation of incident light, and interact with the electromagnetic field to form a near‐field electromagnetic wave localized on the metal surface, which realizes immensely localized electromagnetic field enhanced. When the target microRNA molecules are captured by the fixed TDN carriers in the enhanced electromagnetic field, the fingerprint information of the target microRNA molecule is detected by the MPA through their coupling. Then the information of the target molecule is acquired by observing the spectrum of the absorber. Here, the enhanced field greatly amplifies the infrared vibrations of the molecules, thereby facilitating the observation of extremely low concentrations of target molecules. Noted worthily, the best amplification effect is obtained when the vibration frequency of the target molecule overlaps the resonant frequency of the MPA. Figure 1c shows the spectrum of the MPA‐based biosensor when the microRNA molecule matches the device well. Clearly, the peak at 1665 cm^−1^ representing the stretching vibration of *miR‐155* is observed.

### 2.2 Device Design and Simulation

**Figure 2:**
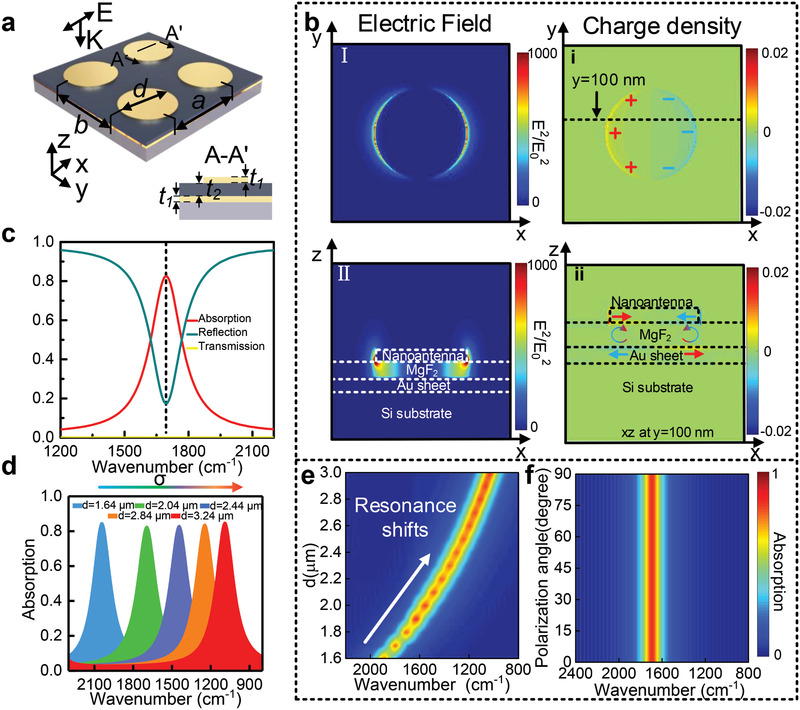
a) Schematic of the MPA, composed of circular Au nanoantennas and an Au sheet separated by an MgF_2_ interlayer, the polarization and propagation direction of the incident light source are illustrated. The array periodicity *a* = *b* = 4 µm, and the diameter *d* = 2.04 µm. b) Electric field enhancement (I, II) and charge intensity (i, ii) in the *xy*‐plane and *xz*‐plane. c) The simulated spectra of the MPA at 1694 cm^−1^. d) Simulated spectra of the MPA with different diameters. e) The relationship between the diameters of nanoantenna and the absorption resonance. f) The simulated spectra of the MPA with different polarization degrees of incident light.

The proposed MPA, as illustrated in **Figure** **2**a, is composed of the bottom Au layer of 100 nm thickness, and the same thick top metallic nanoantennas separated by the dielectric spacer layer MgF_2_ of 200 nm thickness. The metal‐dielectric‐metal Fabry‐Perot cavity is formed by this three‐layer sandwich structure. When the incident infrared light is irradiated on the device surface, it is reflected multiple times and absorbed in the cavity. Perfect absorption is achieved at resonance by rationally optimizing the layer thickness and top pattern (Figure 2a). Absorption *A* of the MPA is calculated as *A* = 1 − reflection *R* − transmission *T*. As the thick *t*_1_ of the ground Au layer is 100 nm, more than the light penetration depth, *T* is nearly zero. Therefore, the absorption spectrum is calculated by the reflectance spectrum as shown in Figure 2c. The optical response of the MPA was studied using 3D finite‐difference time‐domain (FDTD) software. Figure 2bI,II shows the top view (*xy*‐plane) and cross‐sectional view (*xz*‐plane) of the near‐field intensity distribution of the device at absorption resonance, where the incident radiation is *x*‐polarized. In the electric field distribution, the electric field is concentrated around the edge area of the circle, and its maximum enhancement up to 1000. In the charge density distribution (Figure 2bi,ii), the charge on the surface is also concentrated on the edge of the nanoantennas, and the induced charges cause antiparallel currents between the top metal and the bottom metal. As the best amplification effect is obtained when the vibration frequency of the target molecule overlaps the resonant frequency of the MPA, it is necessary to investigate geometry‐dependent characteristics of MPA. Figure 2d shows the simulated spectra of MPA arrays with different diameter *d*. As observed, the resonance wavelength is redshifted with the increase of *d*, which indicates that the device enables to match various molecule vibration by reasonably designing the size of the nanopatterns. Furthermore, we refined the step of the increased radius, and obtain the contour mapping of simulated absorption spectra, as shown in Figure 2e. Clearly, the resonance wavelength is redshifted as the *d* gradually increases from 1.6 to 3.0 µm. This diameter‐dependent is an important feature for the biosensor and provides a simple way to adjust the resonance of MPA, thereby achieving the enhancement of the vibration of the target biomolecule. In addition, the absorber demonstrated polarization‐independent characteristics owing to the omnidirectional symmetry of the designed nanoantennas as exhibited in Figure 2f. Additionally, the near‐field access up to hundreds of nanometers from the dielectric surface with 10% of the near‐field peak intensity (Note S2, Supporting Information).

**Figure 3:**
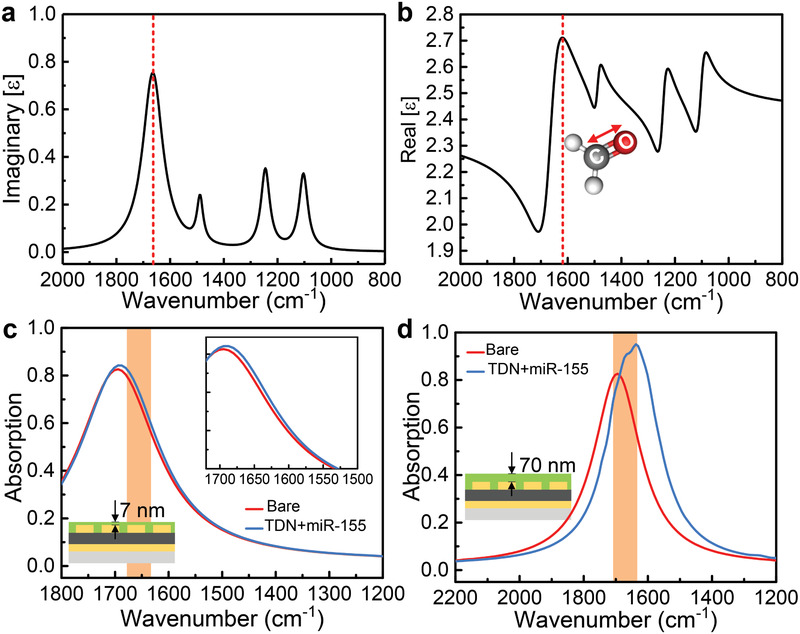
a) Real and b) imaginary parts of the modeled permittivity function for the bilayer composed of TDN and *miR‐155*. The red dashed lines indicate the stretching vibration of the C═O bond around 1665 cm^−1^. c) Simulation spectrum of bare chip and MPA covered by bilayer with a thickness of 7 nm. The inset shows a partial zoom from 1700 to 1500 cm^−1^. d) Simulation spectrum of bare chip and MPA covered by bilayer with a thickness of 70 nm.

To understand the sensing behavior of the biosensor, we simulate the spectral changes of biosensor with the loading of biomolecules. We use the Lorenz model to construct four oscillators to adapt to the infrared reflectance and absorption spectrum of the target biomolecule obtained in the experiment. Its dielectric constant can be written as follows

(1) $$\varepsilon_{\mathrm{bilayer}}=\varepsilon_{\infty }+\sum_{i=1}^{4}S_{i}/\left(\omega_{pi}^{2}-\omega^{2}-j\omega \gamma_{pi}\right)$$

where high‐frequency dielectric constant *ε*_∞_ = 2.37, the oscillator strength, *S*_1_ = 5.01 × 10^26^(rad s^−1^)^2^, *S*_2_ = 5.96 × 10^26^(rad s^−1^)^2^, *S*_3_ = 2.85 × 10^26^(rad s^−1^)^2^, *S*_4_ = 4.06 × 10^27^(rad s^−1^)^2^, the oscillator resonance frequency, *ω*_p1_ = 2.08 × 10^14^(rad s^−1^), *ω*_p2_ = 2.35 × 10^14^(rad s^−1^), *ω*_p3_ = 2.81 × 10^14^(rad s^−1^), *ω*_p4_ = 3.14 × 10^14^(rad s^−1^), and the damping frequency, *γ*_p1_ = 7.54 × 10^12^(rad s^−1^), *γ*_p2_ = 7.53 × 10^12^(rad s^−1^), *γ*_p3_ = 7.30 × 10^12^(rad s^−1^), *γ*_p4_ = 1.72 × 10^13^(rad s^−1^). To simplify the simulation process, the biomolecules adsorbed on the surface of the device are equivalent to a film. **Figure** **3**a,b exhibits the real and imaginary part of the permittivity of the TDN and *miR‐155*, respectively. The red line in Figure 3b indicates the stretching vibration of the C═O bond around 1665 cm^−1^. With the aid of FDTD software, we obtained the spectra of bare MPA device and biomolecule films of different thicknesses, as shown in Figure 3c,d. In Figure 3c, compared with the bare device, the spectrum of the biosensor changes significantly after the loading of the biomolecule layer, including redshift and amplitude change. In addition, as the concentration of biomolecules increases (increase in thickness), the degree of redshift and amplitude change becomes more dramatic (Figure 3d). According to this phenomenon, it indicates that the detection of biomolecules can be achieved by observing changes in the spectrum of biosensor.

### 2.3 Fabrication and Characterization

**Figure 4:**
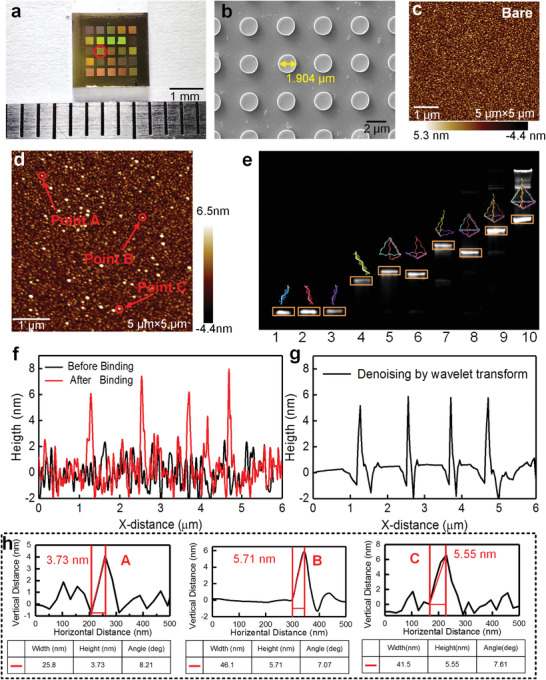
Characterization of MPA and TDN. a) An optical photograph of a MPA chip with 5 × 5 mm^2^ (The sensing area is marked in red). b) Scanning electron microscope (SEM) image of the sensing area. c,d) Atomic force microscopy images before/after the binding of TDN probes. e) Polyacrylamide gel electrophoresis (PAGE) image of TDN. Lane 1: S1, lane 2: S2, lane 3: S3, lane 4: S4, lane 5: S1+S2, lane 6: S2+S3, lane 7: S3+S4, lane 8: S1+S2+S3, lane 9: S2+S3+S4, lane 10: S1+S2+S3+S4. S1: ssDNA1, S2: ssDNA2, S3: ssDNA3, S4: ssDNA4. f) AFM height profile of the surface before and after the binding of TDN probes. g) AFM height profile after denoising using the wavelet transform algorithm. h) Height profiles of the TDN marked by the arrow in (d).

The biosensor was fabricated on a 6‐in. silicon wafer by complementary metal–oxide–semiconductor compatible process, including electron beam evaporation, stepper photolithography, and ion beam etch (IBE) (Note S3, Supporting Information). **Figure** **4**a exhibits an optical photograph of the entire chip with dimensions of 5 × 5 mm^2^, and the sensing area is only 500 × 500 µm^2^, which extremely contributes to miniaturized sensing detection. Additionally, 25 sensing arrays were integrated on the 5 × 5 mm^2^ chip, and nearly 500 such chips were fabricated on a 6‐in. silicon wafer. It indicates we can obtain almost 12 500 sensing arrays by one fabrication. It greatly reduces the cost of the device, making the technology more competitive in sensing applications. Scanning electron microscope (SEM) image of the sensing area is demonstrated in Figure 4b, the metal nanoantennas have clear and uniform boundaries, illustrating the validity of fabrication method. To observe the binding behavior of TDN probes, two methods are used, namely, electrophoresis and atomic force microscopy (AFM). Figure 4c,d shows the AFM images of the chip with/without the TDN probes anchored to the gold surface, respectively. As observed, there are a lot of white dots in the AFM image of the biosensor with the TDN probes. Then we tried to dig deeper into the data to determine the thickness of the probes. First, a distance of 6 µm on the surface is chosen to determine the roughness. The results reveal that the surface roughness of the gold film obtained by electron beam evaporation is about ±2 nm (Figure 4f), which is a great interference for the measurement of the probe thickness. The fluctuation of the rough surface is regarded as the background noise of the measurement. Then, we use wavelet transform algorithm to denoise. Figure 4g is the result of denoising using the wavelet transform algorithm. Clearly, the interference of the Au surface fluctuation is effectively reduce. Then, we determine the thickness of the probes by using the denoised AFM data (Figure 4h). According to the height difference analysis, the height of the TDN probes is distributed between 3.73 and 5.71 nm, which is close to the theoretical height of 5.02 nm and the theoretical edge length of 6.12 nm.^[^ ^35^ ^]^ Therefore, it could be concluded that these points are caused by the binding of TDN to the gold surface. Furthermore, polyacrylamide gel electrophoresis (PAGE) was used to verify the successful preparation of the TDN probe we used, as described in Figure 4e. Clearly, compared with the rest of the single chains (lane 1–4) or the combination of two (lane 5–7) or three (lane 8–9), the TDN structure (lane 10) has the slowest migration rate, which confirms the successful assembly of TDN. Collectively, all of the above morphological and electrophoresis analyses demonstrated the successful preparation and binding of the TDN.

### 2.4 *miR‐155* Detection Using Plasmonic Biosensor

**Figure 5:**
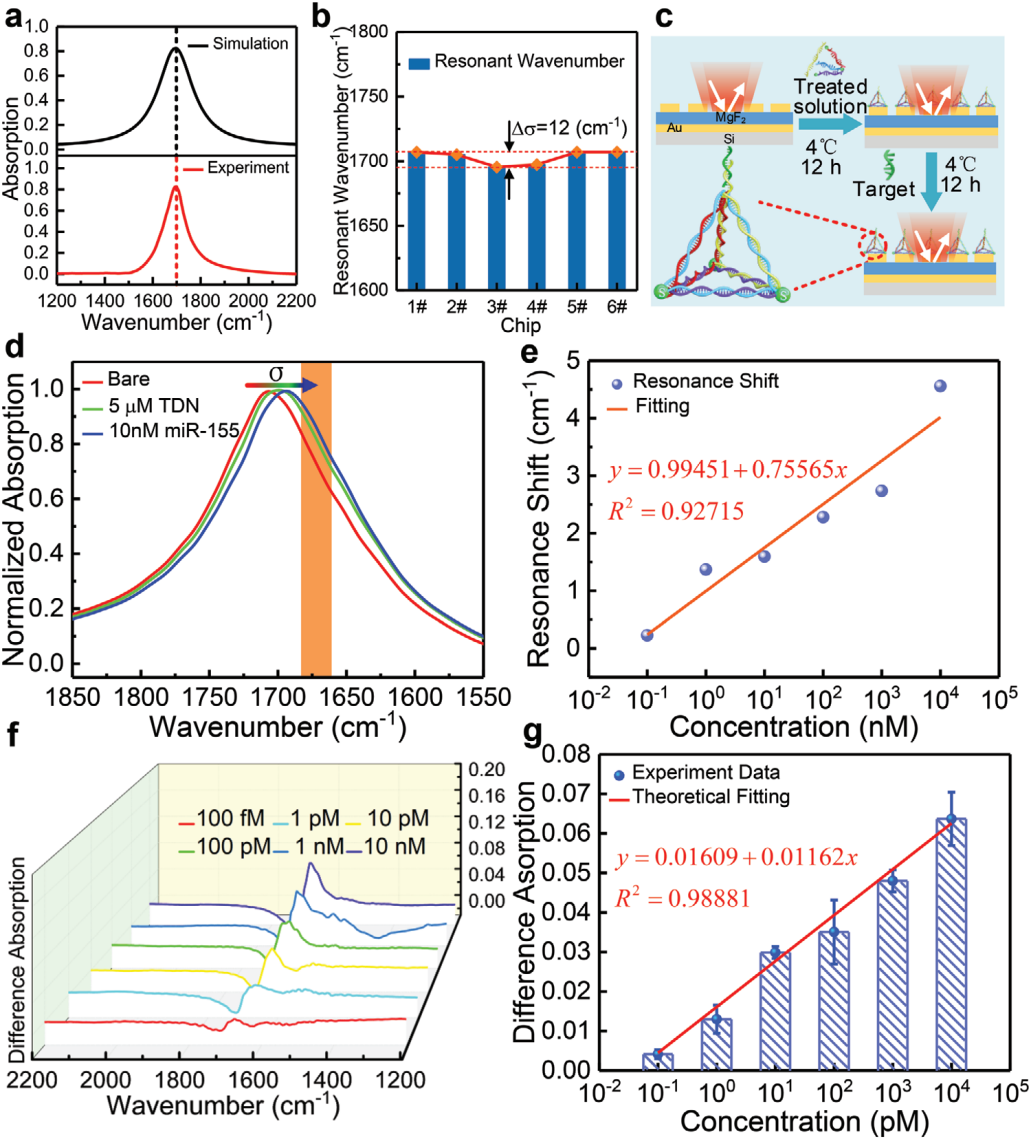
a) Simulated (black) and measurement (red) absorption spectra of the MPA. b) Resonant wavenumber of MPA chips 1# to 6#. c) Experimental preparation procedure. d) Measured normalized absorption spectra of the bare MPA (red), and the MPA after physisorption of TDN (green) and subsequent binding with *miR‐155* with 10 × 10^−9^ m concentration (blue). e) Resonance shift versus concentrations before and after *miR‐155* binding, and fitting to the experimental data using the linear model. f) Difference absorption spectroscopy of different concentrations of *miR‐155*. g) Differential signal as a function of *miR‐155* concentrations, and fitting to the experimental data using the linear model.

Before the detection of *miR‐155*, we first investigate the consistency of the biosensor's spectral response. **Figure** **5**a reveals the simulated and measured absorption spectra of the biosensor. The resonance wavenumber of the simulated spectrum matches well with that of measured spectrum, and both are located near 1700 cm^−1^. Furthermore, six devices fabricated in the same batch (denoted as 1# to 6#) are measured in the same environment, and the results are shown in Figure 5b. Clearly, the resonances are all located near 1700 cm^−1^, and the difference between the maximum (1#) and minimum (3#) wavenumber was 12 cm^−1^, which is only 0.7% of the average wavenumber. These differences are acceptable and can be eliminated by a more sophisticated and expensive fabrication process. The detection of *miR‐155* using the plasmonic biosensor including two processes, namely, device functionalization and *miR‐155* detection, as shown in Figure 5c. In the functionalization process, the treated solution (four ssDNA were dissolved in a buffer to water bath at 95 °C for 10 min) with a concentration of 5 × 10^−6^ m and a volume of 40 µL was dropped onto the surface of the circular gold nanoantennas. After 12 h of incubation at 4 °C, TDN firmly assembled on the surface of the circular metal nanoantennas with the help of Au─S bonds. In the detection process, target *miR‐155* molecules were loaded onto the functionalized biosensor and then left standing at 4 °C for 12 h. The sensor surface was thoroughly rinsed with deionized water between each step and then immediately measured using the IR measurement system. Figure 5d shows the measured results of the biosensor at each step. Two phenomena can be clearly observed, namely, redshift and spectral dip. The resonance redshift is because of the increase of the optical refractive indices in the vicinity of gold nanoantennas after successive binding of biomolecules. In addition, there is a small spectral dip around 1665 cm^−1^, which is consistent with the simulation result in Figure 3c. It is caused by the absorption of C═O stretching vibration in *miR‐155*, demonstrating the previous theory and simulation analysis.

Furthermore, a series of *miR‐155* solutions with various concentrations (100 × 10^−15^, 1 × 10^−12^, 10 × 10^−12^, 100 × 10^−12^, 1 × 10^−9^, 10 × 10^−9^ m) were loaded onto the biosensor to investigate the sensing performance of the biosensor. The detection process is as described above, and the processing and measurement environment are set to be the same. The resonance wavenumber shifts in the presence or absence of *miR‐155* for different concentration cases are shown in Figure 5e, which demonstrates good linear fit results, reflecting the successful binding of *miR‐155* to the absorber surface through TDN scaffolds. The differential signal representing the absorption of C═O stretching vibration in *miR‐155* is extracted by setting the spectrum before *miR‐155* binding as a reference. Figure 5f reveals the extracted difference absorption spectroscopy of *miR‐155* with different concentrations, and the corresponding measured spectra are shown in Note S4 (Supporting Information). As observed, the spectrum changes more drastically as the concentration increases. The detection behavior of the biosensor is further analyzed by the differential spectra and plotting it in Figure 5g. Clearly, the differential signal rises continuously as the *miR‐155* concentration increases, and it can be well fitted by the linear model. The maximum sensitivity of the biosensor detecting *miR‐155* at a low concentration limit can be calculated to be about 1.162% pM^−1^. In addition, the lowest concentration of the target molecule *miR‐155* that can be detected is 100 × 10^−15^ m, indicating the ultrasensitive detection capability of the proposed plasmonic biosensor. Additionally, according to the specificity experiments, the detection is effective when the target molecular *miR‐155* is mixed with *miR‐21* and *miR‐10b* (Note S5, Supporting Information).

### 2.5 Performance Comparison with Other Methods

**Figure 6:**
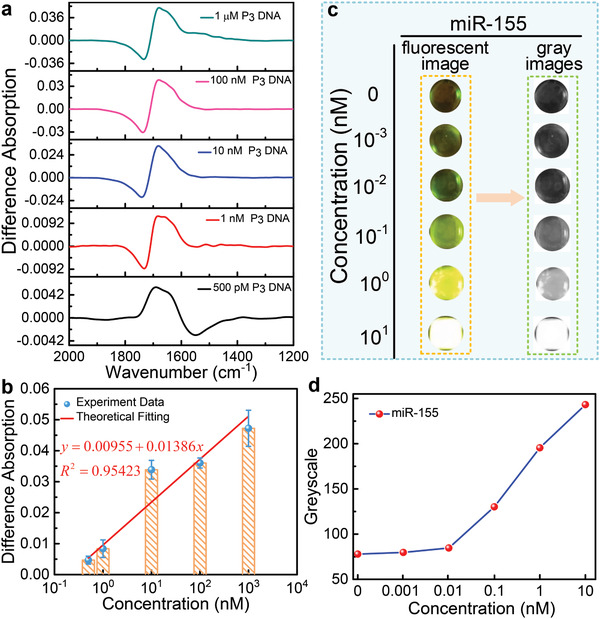
a) Difference absorption spectroscopy of different concentrations of P_3_ DNA. b) Differential signal as a function of P_3_ concentrations, and fitting to the experimental data using the linear model. c) Fluorescence images of different concentrations of *miR‐155*. d) Greyscale for different concentrations in (c).

TDN is not the only carrier for detecting biomolecules, and biosensors with single‐stranded DNA probe can also achieve the detection. For comparison, we conducted an experiment using single‐stranded DNA as probes to detect DNA. The experiment procedure as same as Figure 5c, we replace the TDN with P_1_ DNA to detect the P_3_ DNA under the same treatment condition. The measured spectra are shown in Note S6 (Supporting Information). The difference absorption spectra are extracted to clearly demonstrate the detection signal, as seen in **Figure** **6**a. Figure 6b exhibits the differential absorption signal as a function of P_3_ DNA concentrations, and fitting the experimental data with the linear model. The maximum sensitivity of the biosensor using single‐stranded DNA as probes can be calculated to be about 1.386% nM^−1^, which is 1193 times lower than that using TDN. It is concluded from the above analysis that sensors with TDN as scaffolds is more sensitive than sensors using ssDNA as probes. On the one hand, TDN exhibits excellent structural stability, and the distance between adjacent probes can be controlled. As a result, it is less likely to intertwine, thereby greatly improving the binding efficiency of target molecules. On the other hand, the electrical field strength reaches maximum at a distance of about 0.1 µm from the nanoantennas surface (Note S2, Supporting Information), and the height of the TDN is located at this scale, which results in better molecular enhancement than ssDNA probes.

Detecting microRNA by using fluorescein as a label is a traditional method. The detailed experimental process is described in the Experimental Section. Figure 6c shows the measured results of *miR‐155* detection by using fluorescence method with TDN as probes. As observed, the fluorescence intensity rises with the increase of *miR‐155* concentration. To quantitatively analyze the results, the color fluorescence images are converted to grayscale images, and their intensity is calculated by analysis software. Figure 6d shows the fluorescence intensity as a function of *miR‐155* concentrations. Starting from the concentration of 10 × 10^−12^ m, the fluorescence intensity increased with the increment of *miR‐155* concentration, so 10 × 10^−12^ m was determined as the LOD of this method. Although the LOD of the fluorescence method is up to the picomolar level, owing to the rapid quenching of the fluorescence on the metal surfaces, detection could only be done in liquid environment of *miR‐155*. Therefore, compared with our biosensor, this method suffers from poor detection limits and is complicated to operate because of the need for markers. The comparative analysis of the performance between the SEIRA‐based biosensor and the existing microRNA detection methods is summarized in Table S2 (Supporting Information). Compared to biological methods (such as RT‐PCR and microarray technologies), the SEIRA‐based method is more effective and faster. Compared to fluorescence‐based methods, the SEIRA‐based method is label‐free has a lower LOD. Compared to electrochemical methods, especially electrochemical methods that also use TDN probes, the SEIRA‐based method has a lower LOD but higher stability and repeatability. The LOD of SEIRA‐based method and SPR‐based methods are at the same level. The advantage of SEIRA‐based biosensor is the molecular identification ability, which can be enhanced by extending it to multiplexed detection. Multiresonant SEIRA device is necessary to develop multiplexed detection. Our method for achieving multiple resonances is to add independent resonant units to the existing pattern, as shown in Note S7 (Supporting Information).

## 3 Conclusion

In summary, we have combined metamaterial absorbers with tetrahedral DNA nanostructures to design a novel and elaborate plasmonic biosensor for the detection of *miR‐155* using SEIRA effect. The limit of detection is 100 × 10^−15^ m, about 5000 times lower than DNA single strand as probes and about 100 times lower than the fluorescence detection method, and the C═O bond stretching vibration fingerprint is acquired. In addition, the stability of the designed platform is significantly better than the fluorescence method, which can overcome the fluorescence quenching. The concept is very flexible and can be extended to the detection of various microRNAs or biomolecules through the rational design of MPA and the tetrahedral probe structure.

## 4 Experimental Section

### Numerical Simulations

The spectra, field distribution, charge distribution, and theoretical validation of SEIRA effect were calculated using a commercial software package (FDTD Solutions v8.19, Lumerical Inc.) based on finite‐difference time‐domain method. Periodic boundary condition was used in the *x*‐ and *y*‐directions, perfectly matched layer (PML) boundary condition along the *z*‐direction. The refractive index of Au and Si were derived from Palik et al.,^[^ ^36^ ^]^ and that of MgF_2_ was taken from Dodge.^[^ ^37^ ^]^ The thin titanium (Ti) adhesion layer was omitted from the simulation.

### Fabrication of the MPA

The MPA was fabricated by a complementary metal–oxide–semiconductor compatible process as shown in Note S3 (Supporting Information). A 6‐in. silicon wafer was cleaned and dried as the substrate. Magnetron sputtering technology was used to sequentially deposit the Ti and Au with thickness of 10 and 100 nm, respectively, on the silicon surface. MgF_2_ with 200 nm thick was deposited on the Au layer by an e‐beam evaporator system, Ti (10 nm) and Au (100 nm) were deposited in sequence and then etched by IBE. Here, the Ti acts only as an adhesive layer to enhance the adhesion between the different layers.

### Fourier Transform Infrared (FTIR) Measurements

Infrared absorption spectra were performed using a FTIR spectrometer (IRTracer‐100, Shimadzu) and an IR microscope (AIM‐900, Shimadzu) with a numerical aperture of 0.4 and 15× objective, equipped with mercury cadmium telluride cooled by liquid nitrogen (Note S8, Supporting Information). All data were taken under identical acquisition settings.

4 cm^−1^ resolution, 25 scans co‐added, absorption mode and the knife edge aperture were set to form a measurement area about 100 × 100 µm^2^. All absorption spectra were collected in reference to the absorption of a bare Au mirror.

### Biological Reagents

All ssDNA and *miR‐155* were synthesized by Sangon Biotech. Co., Ltd. (Shanghai, China) and the sequences are listed in Table S1 (Supporting Information). FITC‐labeled primers were purchased from Tsingke Biological technology Co., Ltd. (Beijing, China). DNase/RNase‐free deionized water from TianGen Biotech Co. (Beijing, China) was used to rinse the biosensor in all experiments. The 5× TBE buffer was from Solarbio Science & Technology Co., Ltd. (Beijing, China). The Super GelRed was provided by Takara Bio Co., Ltd. (Beijing, China).

### Fabrication of the Biosensor

TDN was synthesized from three thiolated DNA strands (S1–S3) with 54 nucleotides and 82 nucleotides of S4. S1–S4 with the same concentration of 5 × 10^−6^ m were mixed together and gently stirred for 1 min and in a water bath at 95 °C for 10 min. Afterward, 40 µL volume of the mixture was dropped on the MPA surface and incubated at 4 °C for 12 h to form a stable probe. The infrared spectroscopy was recorded after rinsing the surface with deionized water to remove the unbound TDN. Then, 40 µL of *miR‐155* with various concentrations was captured by TDN under the same condition to acquire the absorption spectroscopy.

### Atomic Force Microscopy

The morphology of bare Au and TDN was observed with atomic force microscopy (Bruker innova, Germany). The samples were scanned in the tapping mode, and the size of TDN was examined using NanoScope Analysis software.

### Native Polyacrylamide Gel Electrophoresis

8% native polyacrylamide gel electrophoresis was performed in 5× TBE buffer. The resulting electrophoresis strips were stained by Super GelRed for 10 min and then imaged for analysis instrument (ChemDoc XR, Bio‐Rad).

### Fluorescence Method

The experimental procedures were same as in the paper except that *miR‐155* was labeled by FITC primer. The fluorescence maps were obtained using Blupad in a liquid environment of *miR‐155* with different concentrations, the Image J software was adopted to acquire fluorescence intensity.

### Statistical Analysis

The spectral data were acquired for the average of 25 scans. The LabSolution (Shimadzu Corporation, Japan) software was used to implement baseline correction and subtraction of spectral data. Wavenumber (*σ*) to wavelength (*λ*) was calculated by*σ* = 10 000/*λ*. Linear regression was used for curve fitting and modeling the relationship between gas concentration and absorption. The linear fitting was performed using Origin software (OriginLab Corporation, USA).

## Conflict of Interest

The authors declare no conflict of interest.

## Supporting information

Supporting Information

## Data Availability

Research data are not shared.
