# Supplementary material for: Infrared Plasmonic Biosensor with Tetrahedral DNA Nanostructure as Carriers for Label‐Free and Ultrasensitive Detection of miR‐155
Source: Adv Sci (Weinh). 2021 Jun 21;8(16):2100583. doi: 10.1002/advs.202100583 (PMC8373097; doi:10.1002/advs.202100583)
Supplement: Supplementary file 1 — Supporting Information [file ADVS-8-2100583-s001.pdf]

© 2021 Wiley-VCH GmbH

ADVANCED SCIENCE
Open Access

Supporting Information

for *Adv. Sci.*, DOI: 10.1002/advs.202100583

# Infrared Plasmonic Biosensor with Tetrahedral DNA Nanostructure as Carriers for Label-Free and Ultrasensitive Detection of *MiR-155*

*Xindan Hui, Cheng Yang, Dongxiao Li, Xianming He, He Huang, Hong Zhou,* Ming Chen, Chengkuo Lee* and Xiaojing Mu**

WILEY-VCH

Copyright WILEY-VCH Verlag GmbH & Co. KGaA, 69469 Weinheim, Germany, 2020.

# Supporting Information

## Infrared Plasmonic Biosensor with Tetrahedral DNA Nanostructure as Carriers for Label-Free and Ultrasensitive Detection of *MiR-155*

*Xindan Hui, Cheng Yang, Dongxiao Li, Xianming He, He Huang, Hong Zhou,* Ming Chen, Chengkuo Lee* and Xiaojing Mu**

# Content

**Supplementary Notes** ........ **3**

Note S1. IR spectroscopy of RNA ........ 3

Note S2. Near-Field penetration depth of the SEIRA platform ........ 4

Note S3. Fabrication process of MPA ........ 5

Note S4. Measured absorption spectra with TDN as probes ........ 6

Note S5. Detection specificity of the SEIRA biosensor ........ 7

Note S6. Measured absorption spectra with ssDNA as probes ........ 8

Note S7. Multiplexed detection ........ 9

Note S8. Instrument platform ........ 11

Table S1. Sequences of DNA and microRNA used in this study ........ 12

Table S2. Performance comparison between the proposed SEIRA-based biosensor and the existing microRNA detection methods ........ 13

**Reference** ........ **15**

# Supplementary Notes

## Note S1. IR spectroscopy of RNA

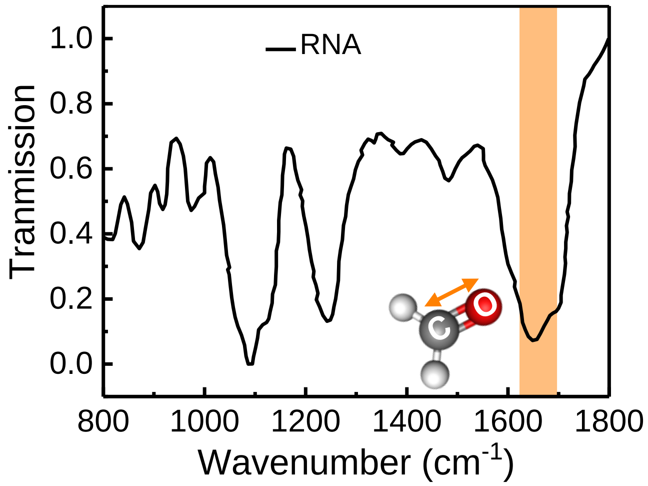


**Figure S1.** The IR spectrum of RNA from 800 $cm^{-1}$ to 1800 $cm^{-1}$, there is a prominent stretching vibration of C=O bond around 1665 $cm^{-1}$.

**Note S2. Near-Field penetration depth of the SEIRA platform**

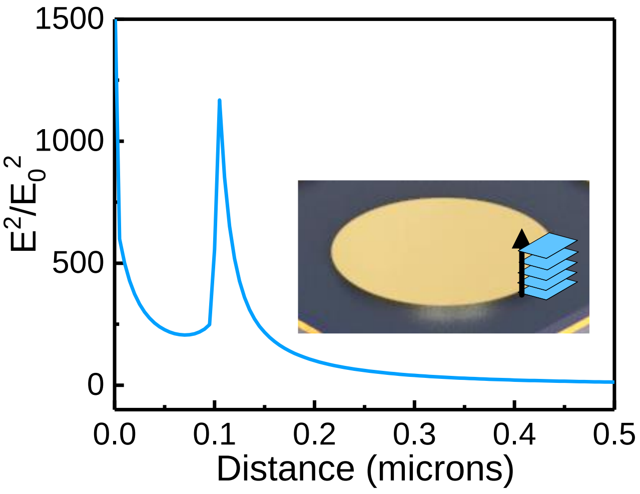


**Figure S2.** The relationship between the electrical field intensity enhancement extending outward and the surface distance. The inset shows the location of the observation point.

**Note S3. Fabrication process of MPA**

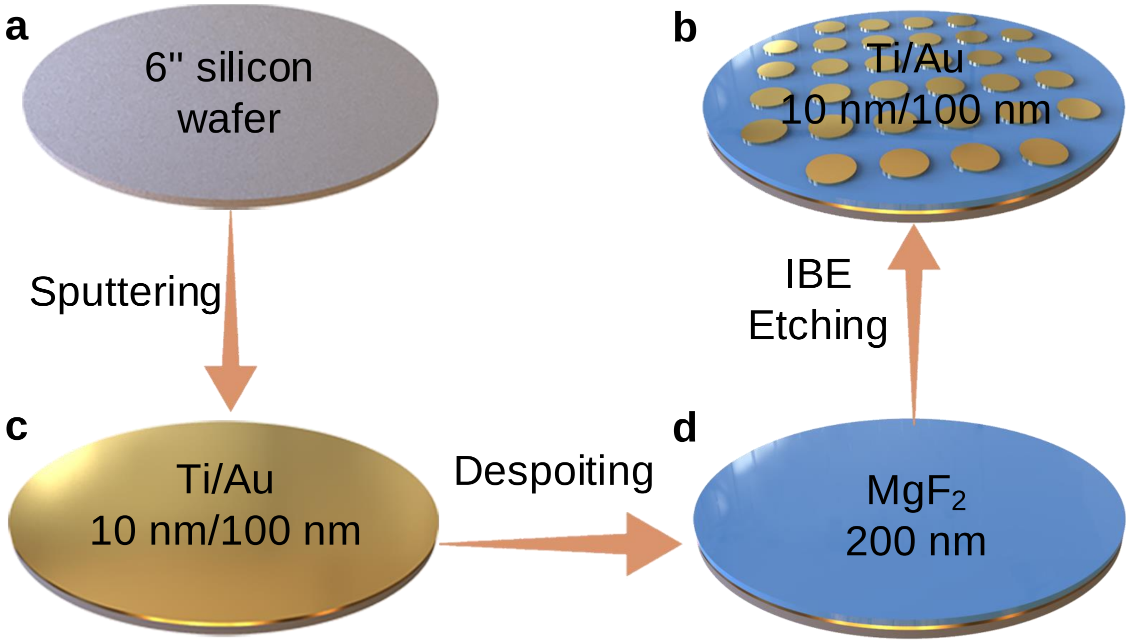


**Figure S3.** Schematic diagram of processes to fabricate the MPA. a) A high-resistivity (20 000) 6" silicon wafer was cleaned and dried for the subsequent use. b) A magnetron sputtering system was used to deposit Ti (10 nm) and Au (100 nm) sequentially on the silicon surface. c) 200 nm thick $MgF_2$ was deposited on the Au layer to form a dielectric layer by an e-beam evaporator system. d) Ti (10 nm) and Au (100 nm) were sequentially deposited on the $MgF_2$ dielectric layer and then etched by IBE to formulate circular nanoantenna.

**Note S4. Measured absorption spectra with TDN as probes**

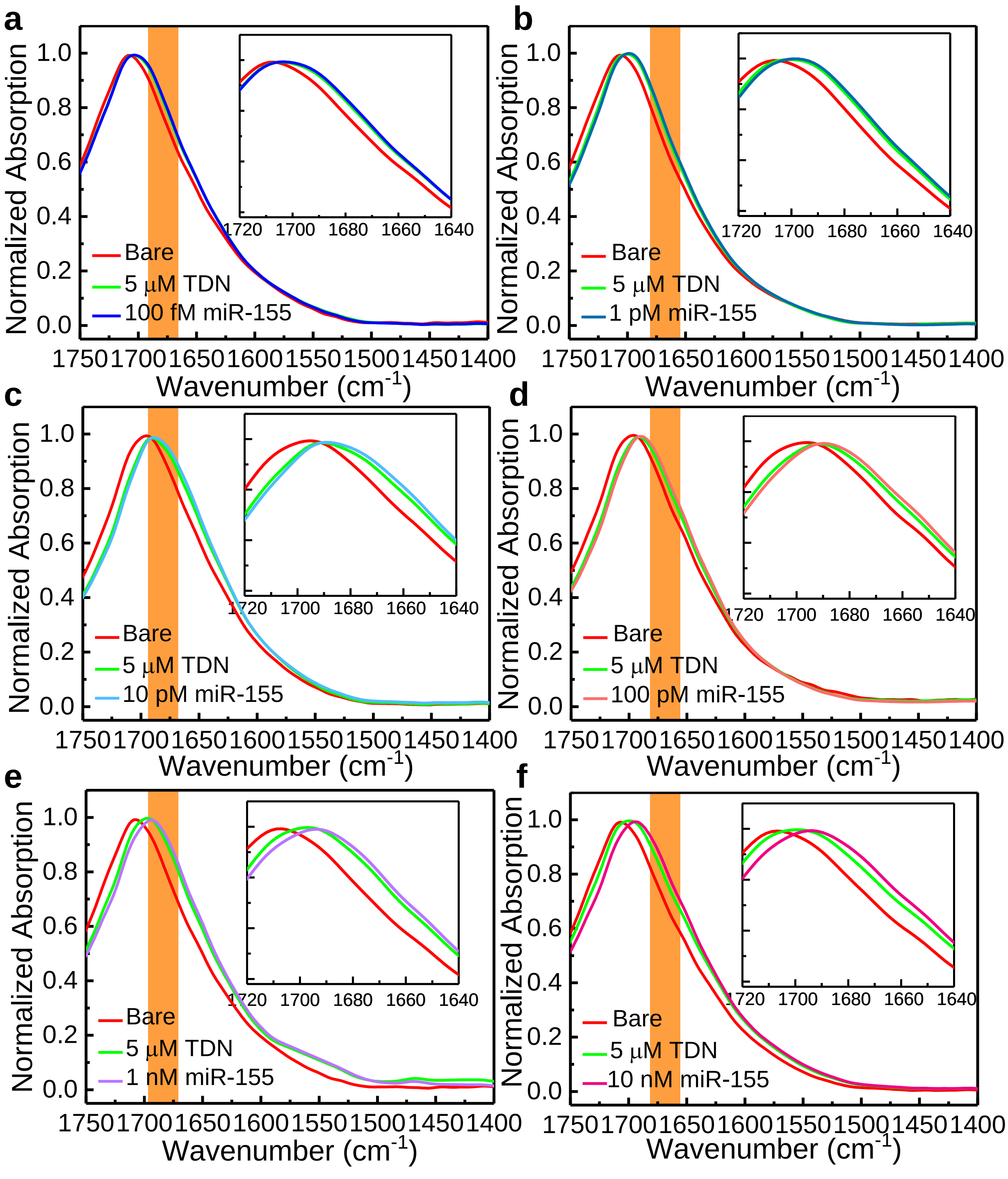


**Figure S4.** a)-f) The spectra of the device after physisorption of TDN probes and subsequent binding with *miR-155* with different concentrations. The shade shows the location of C=O vibration. The inset shows a partial enlargement of wavenumber from 1720 $cm^{-1}$ to 1640 $cm^{-1}$.

**Note S5. Detection specificity of the SEIRA biosensor**

For miRNA sensing, being able to differentiate a single nucleotide polymorphism is important. We have added the experiment on the biosensor's specificity. After the immobilization of the TDN probes, miR-155, miR-21, miR-10b, and their mixture was used as the analytes to investigate the specificity. Their concentration is set to be the same, that is, 10 nM, and the concentration of each component in the mixture is also 10 nM. Figure S5 is the measured result. Clearly, although nonspecific binding is unavoidable, its specificity is effective by using TDN as a probe to sense miRNA.

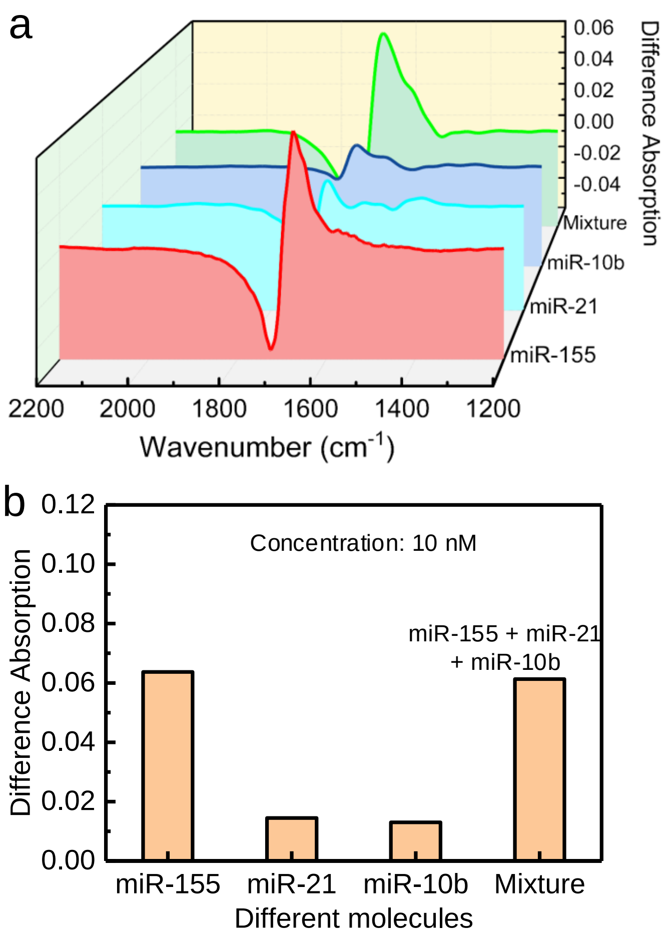


**Figure S5.** Detection specificity of our SEIRA biosensor. a) Differential spectra showing the spectral change of the biosensor after different analytes are loaded. b) Corresponding changes in absorption spectrum at 1700 $cm^{-1}$.

**Note S6. Measured absorption spectra with ssDNA as probes**

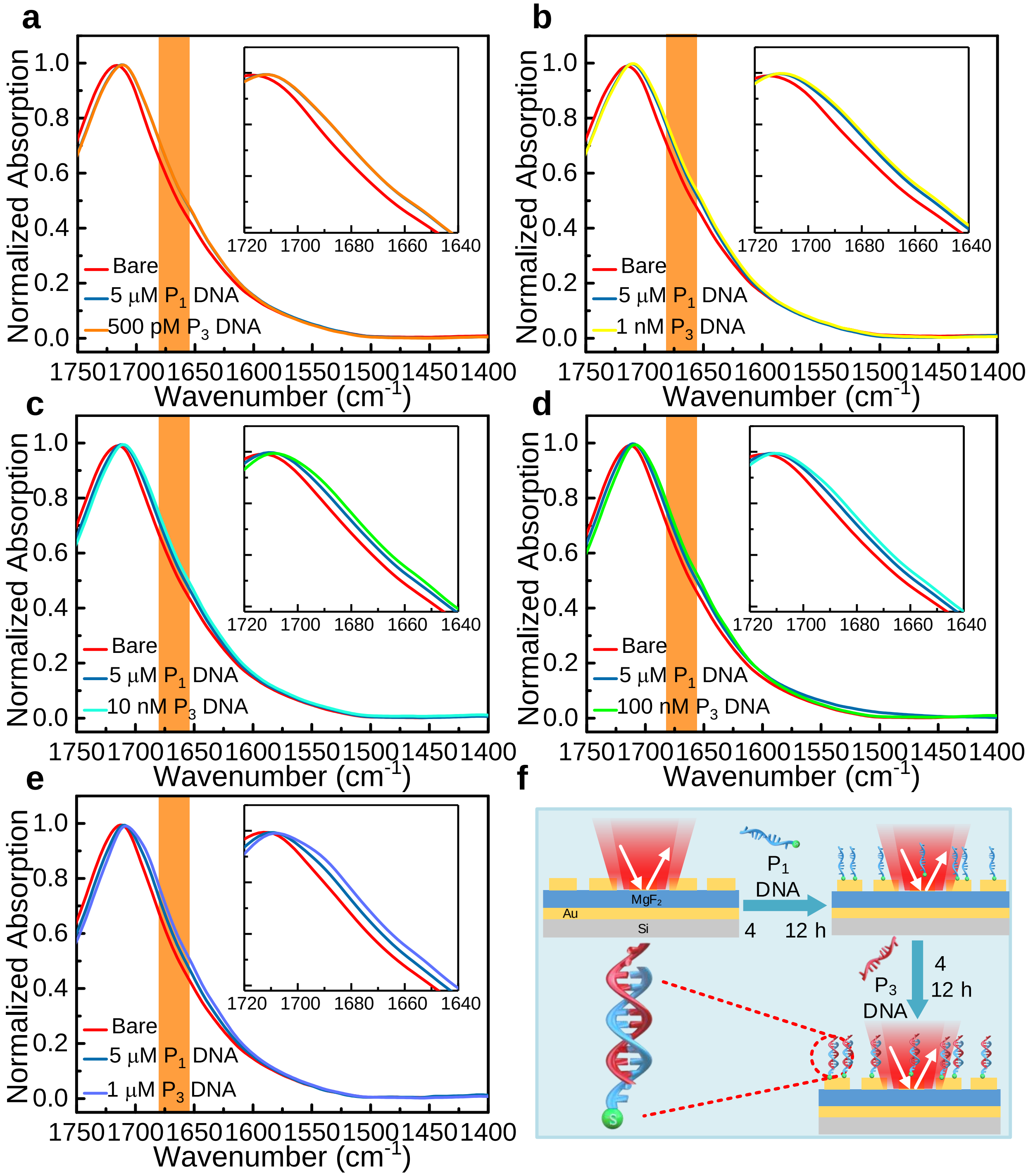


**Figure S6.** a)-e) The spectra of the device after physisorption of $P_1$ DNA probes and subsequent binding with $P_3$ DNA with different concentrations. The shade shows the location of C=O vibration. The inset shows a partial enlargement of wavenumber from 1720 $cm^{-1}$ to 1640 $cm^{-1}$. f) The experimental procedures with $P_1$ DNA as probes.

**Note S7. Multiplexed detection**

To make miRNA sensing eventually translational, it is necessary to look at multiple markers at the same time to improve diagnostic accuracy. The SEIRA technology can be potentially extended to multiplexed detection by developing multi-resonant SEIRA biosensing technology. Each resonance of the multi-resonant SEIRA corresponds to a different IR vibration of multiple markers. Then, multiple markers are detected by observing the spectral change of the resonances of the multi-resonant SEIRA. Our method for achieving multiple resonances is to add independent resonant units to the existing pattern, as shown in Figure S7. By adding two sets of nanodipoles to the disc pattern, the SEIRA device can provide dual (Figure S7a) and triple (Figure S7b) resonances for multiplexed detection. In addition, these resonances is flexible and can be elaborately designed to match the IR vibrations of multiple markers by adjusting the pattern dimension. Resonance A corresponds to the stretching vibration of C=O. Resonance B corresponds to the antisymmetric stretching vibration of $[PO_2]^-$, and resonance C corresponds to the stretching vibration of C-O.

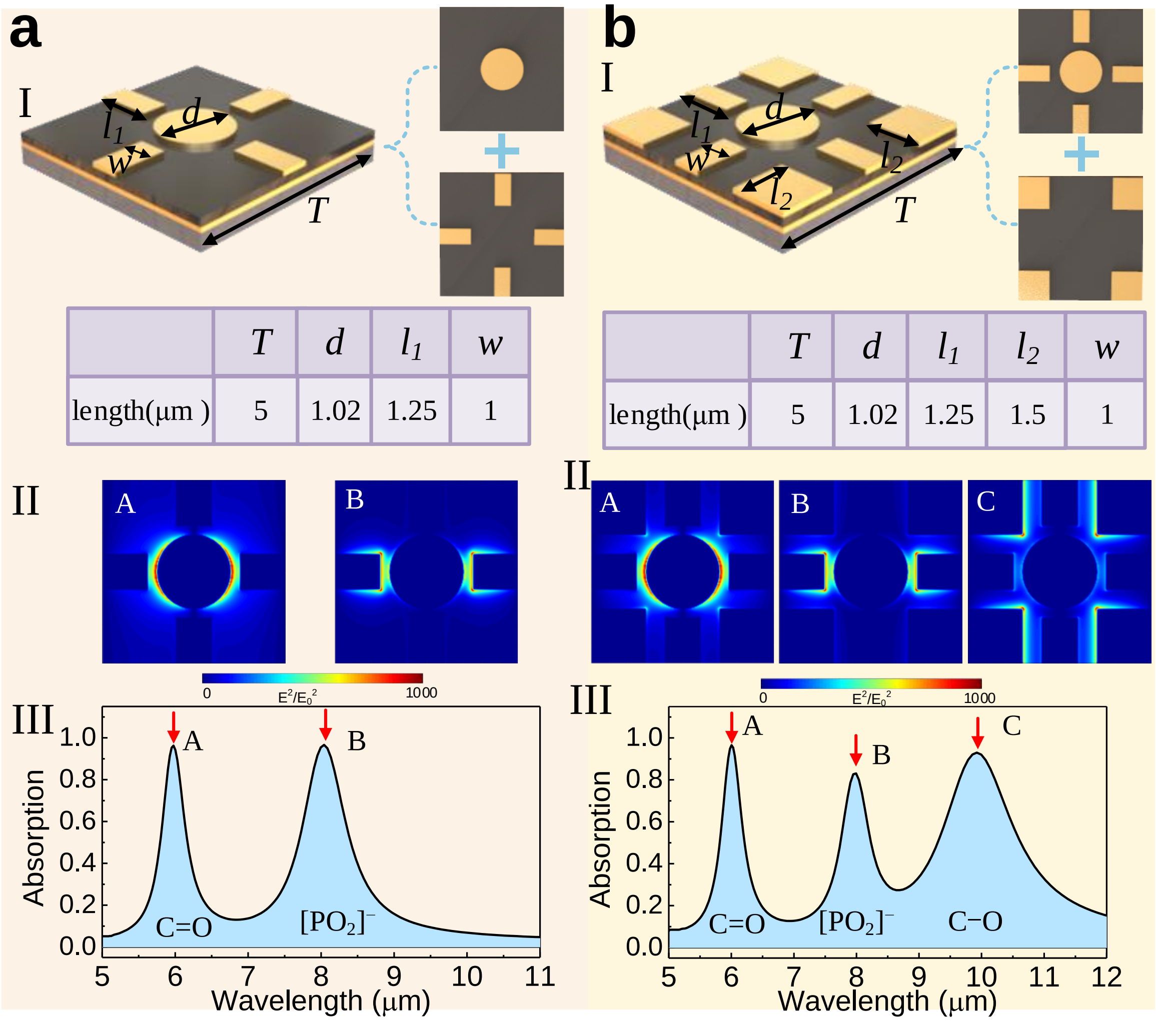


**Figure S7.** Multiplexed detection of multi-resonant SEIRA biosensor. a) Dual-band SEIRA device. b) Tri-band SEIRA device. I: Schematic of the metamaterial absorber. II: Near-field distribution at resonance. III: Simulated absorption spectra. Resonance A corresponds to the stretching vibration of C=O. Resonance B corresponds to the antisymmetric stretching vibration of $[PO_2]^-$, and resonance C corresponds to the stretching vibration of C-O.

**Note S8. Instrument platform**

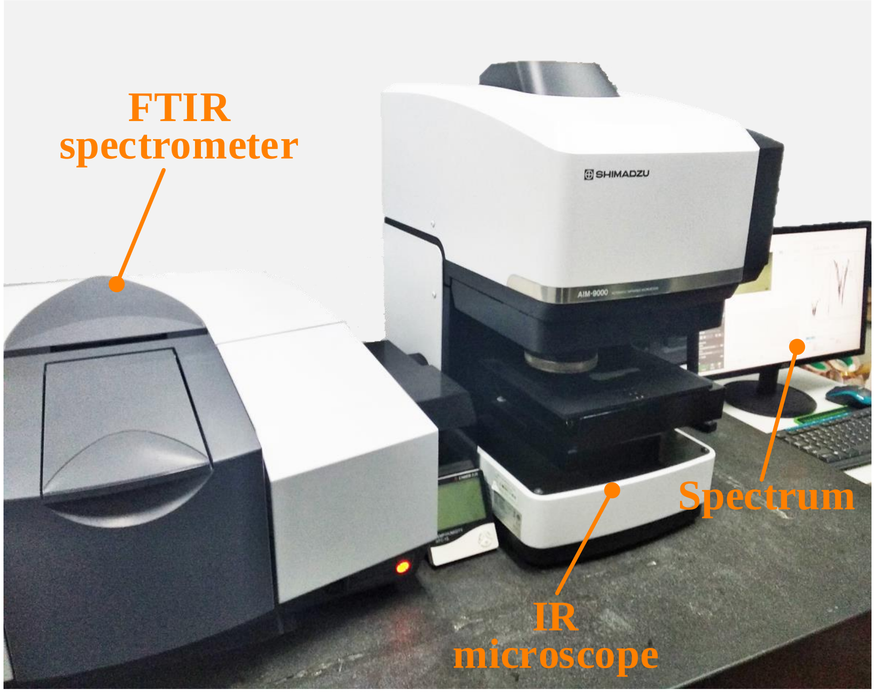


**Figure S8.** The instrument platform of spectral detection.

**Table S1. Sequences of DNA and microRNA used in this study**

| Name | Sequence (5' end to 3'end) | Length(mer) |
|---|---|---|
| **S1** | GCACATTCCTAAGCTGAAACATTACAGCTTGCTACAAGAAGAGCCGCCATAGTA | 54 |
| **S2** | TTATCACCAGGCAGTTGATGTAGCAAGCTGTAATAGATGCGAGGGTCCAATACA | 54 |
| **S3** | TTCAGCTTAGGAATGTGCTTCCCACGTAGTGTCGTTTGTATTGGACCCTCGCAT | 54 |
| **S4** | TCAACTGCCTGGTGATAAAACGACACTACGTGGGAATACTATGGCGGCTCTTCTTTTTACCCCTATCACGATTAGCATTAA | 82 |
| ***miR-155*** | TTAATGCTAATCGTGATAGGGGT | 23 |
| ***miR-10b*** | UACCCUGUAGAACCGAAUUUGUG | 23 |
| ***miR-21*** | UAGCUUAUCAGACUGAUGUUGA | 22 |
| **$P_1$ DNA** | GCACATTCCTAAGCTGAAACATTACAGCTTGCTACAAGAAGAGCCGCCATAGTA | 54 |
| **$P_3$ DNA** | TTCAGCTTAGGAATGTGCTTCCCACGTAGTGTCGTTTGTATTGGACCCTCGCAT | 54 |

**Table S2. Performance comparison between the proposed SEIRA-based biosensor and the existing microRNA detection methods**

| Reference | Probes | Target molecule | Readout Methods | Remarks | LOD |
|---|---|---|---|---|---|
| Schmittgen et al., 2004[1] | / | *miR-21 et al.* | RT-PCR methods | High sensitivity;<br>Time-consuming; Complicated preparation procedure; | / |
| Mattie et al., 2006[2] | Immobilized oligonucleotide probes | *miR-21 et al.* | Microarray technology | High throughput; High sensitivity;<br>Time-consuming; Complicated preparation procedure; | pg level |
| Yue et al., 2019[3] | bCHA-DNA | *miR-155* | Fluorescence-based biosensor | Enzyme-free; Dynamic monitoring; Good selectivity;<br>Poor sensitivity; Fluorescent label required; | 2.5nM |
| Li *et al.,* 2018[4] | DNA-AuNPs | *miR-155* | DNAzyme-based amplification strategy | High selectivity and accuracy;<br>Rapid and effective; Fluorescence quenching;<br>Fluorescent label required; | 44 pM |
| Zhen et al., 2017[5] | TAMRA-DNA-GO | *miR-21* | Fluorescence-based biosensor | Simple; Cost-effective; High selectivity; Poor sensitivity;<br>Low stability; Fluorescence quenching;<br>Requires fluorophore modifications; | 47 pM |
| Chai *et al.,* 2019[6] | QD-DNA-AuBHQ probe | *miR-21* | Fluorescence-labeled nanoparticles | High selectivity; Low background signal;<br>Good biocompatibility; Poor sensitivity;<br>Fluorescence quenching; Fluorescent label High stability | 0.22 nM |
| Liu *et al.,* 2020[7] | AuNRs, AuNP and hairpin DNA | *miR- 21* | Dark-field microscope | Low background signal; High spatial imaging;<br>High discrimination; Poor sensitivity; Polarization dependent;<br>Complicated preparation procedure; | 2 pM |

| Reference | Probes | Target molecule | Readout Methods | Remarks | LOD |
|---|---|---|---|---|---|
| Lu et al., 2019[8] | TDN probes | *miR-21* | Electrochemistry biosensor | Satisfactory sensitivity; High selectivity; Acceptable accuracy; Requires nuclease modification; Complicated experimental procedures; | 0.04 fM. |
| Wang *et al.,* 2017[9] | Y-shaped ds-DNA | *miR-21* | Electrochemistry biosensor | High sensitivity; High selectivity; Requires multiple aptamers; Cumbersome experimental procedures; Time-consuming; | 10 aM |
| Fan *et al.*, 2012[10] | TDN probes | *miR-21* | Electrochemistry biosensor | High sensitivity; High selectivity; Good accuracy; Sensitive to temperature *et al.*; | 10 aM |
| Aoki *et al.*, 2019[11] | DNA-$NH_2$ | *miR-17* *miR-21* | surface plasmon resonance (SPR)-based biosenor | Simple, cost-effective, and feasible; Expensive SPR imager required; | 0.5 pM |
| This work | TDN probes | *miR-155* | SEIRA-based biosensor | Label-free; Simple; High sensitivity; High stability Cost effectivity; Not sensitive to temperature; Infrared spectrometer required; | 100 fM |

## Reference

[1] T. D. Schmittgen, J. Jiang, Q. Liu, L. Yang, *Nucleic Acids Res.* 2004, 32, e43.

[2] M. D. Mattie, C. C. Benz, J. Bowers, K. Sensinger, L. Wong, G. K. Scott, V. Fedele, D. Ginzinger, R. Getts, C. Haqq, *Mol. Cancer* **2006**, 5, 24.

[3] S. Yue, X. Song, W. Song, S. Bi, *Chem Sci* **2019**, 10, 1651.

[4] P. Li, M. Wei, F. Zhang, J. Su, W. Wei, Y. Zhang, S. Liu, *ACS Appl. Mater. Interfaces* **2018**, *10*, 43405.

[5] S. J. Zhen; X. Xiao, C. H. Li, C. Z. Huang, *Anal. Chem.* **2017**, 89, 8766-877

[6] S. Q. Chai, W. Y. Lv, J. H. He, C. H. Li, Y. F. Li, C. M. Li, C. Z. Huang, *Anal Chem* **2019**, *91*, 6761.

[7] J. J. Liu, H. H. Yan, Q. Zhang, P. F. Gao, C. M. Li, G. L. Liang, C. Z. Huang, J. Wang, *Anal. Chem.* **2020**, *92*, 13118.

[8] J. Lu, J. Wang, X. Hu, E. Gyimah, S. Yakubu, K. Wang, X. Wu, Z. Zhang, *Anal. Chem.* **2019**, 91, 7353.

[9] Y. Wang, X. Zhang, L. Zhao, T. Bao, W. Wen, X. Zhang, S. Wang, *Biosens. Bioelectron.* **2017**, *98*, 386.

[10] Y. Wen, H. Pei, Y. Shen, J. Xi, M. Lin, N. Lu, X. Shen, J. Li, C. Fan, *Sci Rep* **2012**, 2, 867.

[11] H. Aoki, R. M. Corn, B. Matthews, *Biosens. Bioelectron.* **2019**, 142, 111565.
